# First records of *Synoeca septentrionalis* Richards, 1978 (Hymenoptera, Vespidae, Epiponini) in the Brazilian Atlantic Rain Forest

**DOI:** 10.3897/zookeys.151.1882

**Published:** 2011-12-03

**Authors:** Rodolpho S. T. Menezes, Sergio R. Andena, Antonio F. Carvalho, Marco A. Costa

**Affiliations:** 1 Universidade Estadual de Santa Cruz, Departamento de Ciências Biológicas, Rod Ilhéus/Itabuna Km 16, Ilhéus, Bahia, Brazil, 45650-000; 2 Universidade Estadual de Feira de Santana, Departamento de Ciências Biológicas. Av Transnordestina, s/n, Feira de Santana, Bahia, Brazil, 44036-900

**Keywords:** wasps, geographic distribution, *Synoeca*

## Abstract

Nests of *Synoeca septentrionalis* were collected in two Brazilian Atlantic Rain Forest localities (Itabuna and Santa Terezinha, in the state of Bahia and Alfredo Chaves in the state of Espírito Santo). *Synoeca septentrionalis* was previously recorded only from Central America and northwestern South America. This findingextends its geographical distribution to Northeast and Southeast regions of Brazil, and represents the first record for *Synoeca septentrionalis* in the Brazilian Atlantic Rain forest, raising to three the number of *Synoeca* species known from Bahia State.

## Introduction

*Synoeca* is a small genus of social wasps, with five species described, widely distributed in Central and South America (Richards 1978; Carpenter and Marques 2001). The nest architecture is similar in all species: arboreal, usually on a broad slanting surface and with a single sessile comb attached directly to the tree trunk (Wenzel 1998). The genus was recently subject of a phylogenetic analysis, being supported as monophyletic (Andena et al. 2009).

Although four species of *Synoeca* have been recorded in Brazil, *Synoeca septentrionalis* was previously recorded only from North and Central America (Mexico, Guatemala, Belize, Honduras, El Salvador, Costa Rica, Nicaragua and Panama) and northwestern South America (Colombia, Venezuela, Ecuador, Peru and Bolivia) (Richards 1978; Andena et al. 2009; Cely and Sarmiento 2011). Active nests of *Synoeca septentrionalis* were discovered and collectedin Atlantic Rain Forest in Itabuna (14°47'S, 39°16'W) and Santa Terezinha (12°46'S, 39°31'W), Bahia State, and in Alfredo Chaves (20°38'S, 40°45'W), Espírito Santo State.

**Figure 1.**
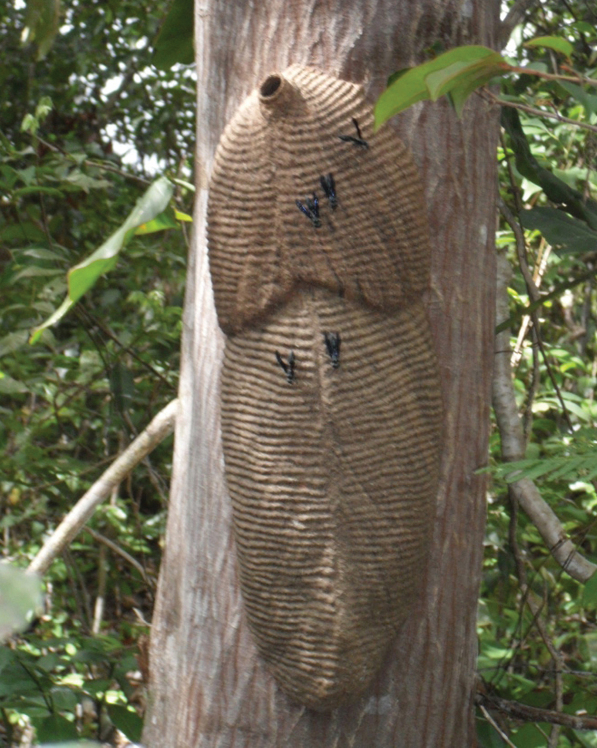
Nest of *Synoeca septentrionalis* collected in Santa Terezinha in the state of Bahia.

The nests collected present a longitudinal groove extending from top to bottom, and a central-dorsal ridge (Fig. 1). Richards (1978: 181) reported similar nests features in *Synoeca septentrionalis* collected in Cali (Colombia). The nest of *Synoeca surinama* is similar to *Synoeca septentrionalis* with a central-dorsal ridge (Castellon, 1980: fig. 5). However, nest of *Synoeca surinama* has a keel instead of a groove. The nests were found attached to tree trunks (Santa Terezinha c. 1,70 m from the ground and two combs; Itabuna c. 6 m from the ground and one comb) and attached on the rock (Alfredo Chaves c. 2,10 m from the ground and two combs). Elisei et al. (2005) and Sidnei Mateus (pers. com.) also recorded nests of *Synoeca cyanea* attached on the rock in the states of Minas Gerais and Mato Grosso.

*Synoeca septentrionalis* is easily diagnosed by the presence of the outstanding erect hairs on the first metasomal tergum and sternum and by a dark triangular area in clypeus (Richards 1978; Andena et al. 2009). However, variation in the clypeus color pattern was observed. The nest collected in Santa Terezinha had eight females with clypeus entirely reddish, two females with clypeus reddish with a dorsal dark area, eight males with clypeus reddish with a triangular dorsal dark area, and five males with the clypeus reddish with a dark dorsal area. The specimens collected in Alfredo Chaves (eigth females) and Itabuna (14 females) had the clypeus entirely reddish. Richards (1978: 181) also reported variation in clypeus color in specimens collected in Ecuador and Colombia.

**Figure 2.**
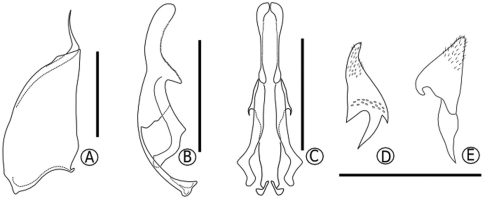
Male genitalia: **A** paramere **B** aedeagus in lateral view **C** aedeagus in ventral view **D** digitus in lateral view **E** cuspis in lateral view. Scale Bar = 0.5 mm.

Voucher specimens are deposited at Entomological Collections at the Universidade Estadual de Santa Cruz, Ilhéus, Brazil and at the Universidade Estadual de Feira de Santana (MZUEFS), Feira de Santana, Brazil. Despite the male genitalia have been cited by Richards (1978) and used in a phylogenetic analysis by Andena et al. (2009) we also provided detailed drawings of this structure (Fig. 2).

This finding extends geographical distribution of *Synoeca septentrionalis* to Northeast and Southeast regions of Brazil, and represents the first record for *Synoeca septentrionalis* in the Brazilian Atlantic Rain forest, raising to three the number of *Synoeca* species known from Bahia State, *Synoeca cyanea* (Richards 1978; Santos et al. 2007), *Synoeca surinama* (Richards 1978), and *Synoeca septentrionalis*.

Geographic distribution of other Epiponini, *Epipona media* Cooper, previously described to range from Ecuador, Peru and Brazilian Amazon to the state of Goiás was recently extended to Atlantic Rain Forest by Menezes et al. (2010). These data suggest that more exhaustive sampling in this region are needed to provide a diagnosis of the distribution of species, and a frame of the diversity in the biome of Atlantic Rain Forest for its preservation.
